# Unraveling Persistent Genital Arousal Disorder—A Case Report on Innovative Therapeutic Approaches

**DOI:** 10.1002/ccr3.72547

**Published:** 2026-04-15

**Authors:** Abdullah Hassan, Arooma sagheer, Muhammad Khubaib Ullah, Habib Ullah, Bilal Hassan, Sardar Noman Qayyum, Rafiullah Hotak

**Affiliations:** ^1^ Ayub Medical College/Ayub Teaching Hospital Abbottabad Pakistan; ^2^ Ayub Teaching Hospital Abbottabad Pakistan; ^3^ Peshawar Medical College Peshawar Pakistan; ^4^ MTI, Ayub Teaching Hospital Abbottabad Pakistan; ^5^ Bacha Khan Medical College Mardan Pakistan; ^6^ Nangarhar University Faculty of Medicine Jalalabad Nangarhar Afghanistan

**Keywords:** family planning/reproductive health, mental health, obstetrics/gynecology, psychiatry

## Abstract

Persistent genital arousal disorder (PGAD) is a rare and distressing condition characterized by persistent, unwanted genital arousal in the absence of sexual desire, often leading to significant psychological distress and functional impairment. We present the case of a married woman in her 30s with a 5 month history of spontaneous genital tingling, throbbing, and warmth that was exacerbated by sitting and not associated with sexual thoughts or behaviors. Her symptoms were accompanied by anxiety, depressive features, sleep disturbance, and social withdrawal. The patient had a significant psychosocial history, including early parental loss, multiple miscarriages, and emotional distress related to infertility and family responsibilities. Investigations revealed a hyperdense focus in the right parietal lobe on computed tomography, while magnetic resonance imaging and electroencephalography were unremarkable. Pelvic imaging demonstrated a bicornuate uterus. Psychometric evaluation indicated moderate anxiety and depression. Multiple prior pharmacological treatments yielded minimal or temporary benefit. A multidisciplinary treatment approach was initiated, including risperidone, sodium valproate, fluoxetine, and short‐term clonazepam, alongside structured psychotherapy focusing on relaxation techniques and cognitive behavioral strategies. Significant clinical improvement was observed within 1 week, with marked reduction in symptoms and improved overall functioning. This case highlights the multifactorial etiology of PGAD, involving neurophysiological, reproductive, and psychological factors. It underscores the importance of a comprehensive biopsychosocial approach, where combined pharmacotherapy and psychotherapy can lead to meaningful symptom relief and improved quality of life in affected individuals.

## Key Clinical Message

Persistent Genital Arousal Disorder is an unpleasant, underdiagnosed illness that requires a collaborative approach. This instance demonstrates the significance of simultaneously treating neurophysiological, psychosocial, and reproductive issues. A customized regimen of Risperidone, Sodium Valproate, and CBT considerably reduced symptoms, highlighting the importance of individualized, integrated care in complex psychosexual diseases.

## 1 Introduction

Persistent genital arousal disorder (PGAD) is a pathological condition characterized by persistent, unwanted sexual arousal associated with the sensation of genital tingling, throbbing, pulsating, and pressure in the absence of any conscious sexual desire or interest that may or may not have identifiable etiology. PGAD may be associated with the feeling that orgasm is impending and symptoms are either not diminished after orgasm or there is only temporary relief postorgasm. This can be potentially debilitating, contributing to significant discomfort. Leiblum and Nathan were the first to report this condition in 2001 after conducting a case series of five female patients. They first coined the term “persistent sexual arousal syndrome” [1] which was later replaced as PGAD by Leiblum in 2006 since “the condition was more a problem of genital than sexual arousal” [2]. PGAD is a distinct condition, not to be misdiagnosed with hypersexuality disorders, which present with spontaneous uncontrolled sexual urges resulting in repetitive sexual behavior [3]. PGAD patients have been reported to suffer from comorbid depression, anxiety, and suicidal ideation [4]. Since there is limited clinical research and data analysis of PGAD, little is known about its pathophysiology, and the majority of information is derived from case reports from around the world. According to the International Society for the Study of Women's Sexual Health (ISSWSH), a regional diagnosis algorithm has been developed that divides the pathophysiology of PGAD into five regions [5]. The first region to consider is the end organ where the cause can be clitoral, vestibular, bladder, vulvar, or vaginal pathology [5, 6]. The second region involves perineal/pelvic causes such as hypertonic pelvic floor muscle dysfunction, pudendal nerve pathology, and arterial–venous malformations [5, 7]. The third region is cauda equina, where PGAD may be associated with Tarlov cyst [5, 8]. In the spinal cord, the fourth region, we speculate that PGAD symptoms are due to issues like disc herniation or annular tear [5, 9]. The fifth region is the brain, where causes can include discontinuation or abrupt initiation of CNS medications such as antipsychotics, anticonvulsants, and antidepressants [5, 10]. Organic brain pathologies associated with PGAD include head injury, epileptic seizures, etc. [5, 11] In addition to these organic factors, are psychological factors that contribute to the development of PGAD which were previously overshadowed [5, 12]. Notably, patients with PGAD have significantly reported of comorbid depression and anxiety, leading to unsatisfactory social and sexual life [13].

We present a case of a woman in her 30s who presented in outpatient department (OPD) complaining of genital tingling and throbbing sensation along with headache. This case report outlines the clinical presentation, radiological findings, and psychological and pharmacological management of the patient.

## 2 Case Presentation/History

The patient is a heterosexual married woman in her 30s who presented to the outpatient clinic of our hospital with a 5 month history of spontaneous, distressing, unwanted genital arousal. She described the symptoms as a feeling of tingling, throbbing, and warmth in her genital area that occasionally radiates to her leg. Notably, these symptoms were not painful, scoring 0 on the visual analog scale (VAS). The severity was largely mild but fluctuated to moderate or severe intensity. She reported experiencing these symptoms throughout the day amidst her routine activities. Importantly, there was no report of sexual desire or fantasy associated with these episodes. The patient did not engage in masturbation for the relief of these symptoms. The arousal was exacerbated by sitting and was initially associated with urinary frequency and urgency. Additionally, the patient had a comorbid severe headache that was sharp in character and radiated across her head but it resolved within days while the genital sensations persisted.

The patient mentions that the impact of the disease on her daily life has been profound. She had been increasingly preoccupied with her condition, slowly developing anxiety, irritability, and sadness. She reported difficulty concentrating, losing sleep, and experiencing social withdrawal. She lost interest in the day‐to‐day activities she previously enjoyed such as cooking. According to the patient, her husband, who served in the military, was deployed for 6 months in the past year. During this time, she became sexually frustrated. Exhausted and burdened by guilt, she lost interest in sexual activities with her husband, but she did not completely abstain from it.

The patient comes from a traditional South Asian background. She endured a challenging life, marked by the death of her father, an army officer, at the age of 14 years, followed by her mother at 17. After the death of her parents, she and her younger siblings were brought up by her uncle. She left her education early in 10th grade to help with household chores. She got married to her current husband at the age of 18 years old. She started having heterosexual relations with her husband but could not conceive, leading to six miscarriages. She ultimately adopted her brother‐in‐law's 2 days old newborn son to whom she is deeply attached. Despite support from family, she is anxious about her ability to raise her son due to her condition.

## 3 Differential Diagnosis, Investigations and Treatment

The patient presented with a CT scan of the brain showing hyperdense focus in the right parietal lobe adjacent to the right frontal horn. On further investigations, an MRI of the brain was done which was unremarkable. MRI and the ultrasound of the pelvic region gave the impression of a bicornuate uterus. Her EEG showed no abnormal activity. Gynecological examination revealed no anatomical abnormalities, clitoral overexposure, vaginal engorgement, or scarring. Her beta HCG came back positive with a value > 5000 mLU/mL at fourth week of gestation. She was diagnosed with twin pregnancy but later had her seventh miscarriage while admitted to our hospital. As a part of routine screening, the patient was tested for sexually transmitted diseases, including syphilis using VDRL (Venereal Disease Research Laboratory test) and HIV through fourth generation antigen–antibody assay, all of which came back negative. For psychometric evaluation, the Hamilton Anxiety Rating Scale (HAM‐A) and the Hamilton Depression Rating Scale (HDRS) were applied, scoring 18 and 21, respectively.

The patient's previous treatment history included the use of a half tablet of Quetiapine 25 mg at night and Alprazolam 0.25 mg at night for 1 month which. Despite initial improvement in symptoms, the patient experiences a relapse. Subsequently, treatment with a combination of Olanzapine‐Fluoxetine (6/25 mg) at night and Alprazolam half tablet twice daily for 25 days showed no improvement. She then subsequently took Olanzapine 5 mg once daily, Propranolol 10 mg twice daily, and Clonazepam 2 mg half tablet twice daily but reported no benefit.

After our investigations and detailed assessment, the patient was started on a tailored plan focused on combination therapy including a second‐generation antipsychotic, mood stabilizer, SSRI, and benzodiazepine. The patient was started on Risperidone 2 mg at night, Sodium Valproate 250 mg twice daily, fluoxetine 20 mg once daily, and clonazepam 0.5 mg half tablet at night, which was gradually tapered off over 2 weeks. Concurrently, she was provided with twice‐weekly psychotherapy sessions focused on relaxation techniques to address her distress and anxiety.

## 4 Conclusion and Results (Outcome and Follow‐Up)

Within the first week of treatment, significant improvement was seen highlighting the effectiveness of pharmacological and psychotherapeutic interventions. After confirmation of her pregnancy, a critical adjustment was made to her regimen: Sodium Valproate was replaced with Carbamazepine 200 mg to initially ensure fetal safety. Initially, improvement was seen with Carbamazepine, but after miscarriage the patient's symptoms worsened again. Hence, Sodium Valproate was reintroduced, resulting in renewed improvement in her condition.

Before discharge, the patient's symptoms were minimal with occasional mild episodes. A reassessment through HAM‐A and HDRS was not performed. Substantial clinical improvement, however, was noted in the symptoms of anxiety, depression, sleep disturbance, and functional impairment. The patient has been advised to close outpatient follow‐ups for ongoing monitoring. Long‐term psychotherapy is planned to help her with the navigation of her psychological stressors. The patient and her husband have also been guided to refer her to a gynecologist for further investigation concerning her miscarriages, and they have been advised against attempted conception till the clinical picture becomes clear.

## 5 Discussion

PGAD is a rare and debilitating clinical picture that poses a significant challenge to the therapeutic approaches. This case report adds to the otherwise limited existing literature on this disease, stressing the interplay of pathophysiological and psychosocial elements of the disorder and provides an overview of the possible treatment options.

We know that the etiology of PGAD is multifactorial, including vascular and neurological components. The finding of a hyperdense focus in the right parietal lobe of the patient on the CT brain raises concern about the potential neurovascular contribution. There is evidence in the literature that CNS lesions in the parietal lobe that are involved in sensory integration could disrupt the normal processing of genital sensations, resulting in unwanted, persistent arousal without sexual desire [14].

Although the connection between the bicornuate uterus and PGAD is less direct, it serves more as a significant psychological stressor due to its association with multiple miscarriages. However, this anatomical abnormality may alter and irritate the end‐organ nerve pathways. The uterine malformation could disturb the pelvic innervation leading to hypersensitivity or abnormal signaling in the genital area [15]. As a result, while not a prime cause, the bicornuate uterus may indirectly influence the manifestation of PGAD.

The major stressor targeted in our case was psychosocial. The anxiety, depression, and emotional turmoil from thinking about the disease can maintain and exacerbate the symptoms. As evident from the history of the patient, the loss of her parents at an early age, taking up the role of an adult prematurely, and above all, repeated pregnancy loss became the major psychological factors influencing her disease. Thus, to address these stressors, a comprehensive treatment approach is warranted. Our intervention was two‐fold: psychotherapeutic and pharmacological.

Sodium Valproate and Risperidone are the two major pharmacological agents we explored as potential alleviators of the symptoms of PGAD. Sodium Valproate is recognized as both an anticonvulsant and a mood‐stabilizing agent. It mainly works by increasing the levels and activity of gamma‐aminobutyric acid (GABA), an inhibitory neurotransmitter [16]. Research indicates that the arousal in PGAD may be associated with dysregulation of neurotransmitters, such as dopamine and serotonin [17]. The theory is that by modulating the levels of GABAergic activity, Sodium Valproate helps in balancing the activity of these neurotransmitters, ultimately leading to the reduction of unwanted genital sensations. Risperidone, on the other hand, is a second‐generation antipsychotic drug that works as an antagonist of D2 and 5‐HT2A receptors. Since PGAD has an association with excessive dopaminergic activity, Risperidone may help in decreasing spontaneous and persistent genital arousal by blocking dopamine receptors, thereby reducing the heightened arousal response. Moreover, the capacity of Risperidone to restore inhibitory serotonergic control may contribute to modulating aberrant sexual excitation [18]. Lastly, Sodium Valproate and Risperidone, each with distinct mood‐stabilizing mechanisms, can provide significant anxiolytic effects when used in combination. This dual approach may alleviate the psychological distress that exacerbates PGAD symptoms, offering a more comprehensive therapeutic benefit.

In terms of the psychotherapeutic approach, this case emphasizes how incorporating psychotherapy sessions played a vital role in the patient's overall treatment regimen. By incorporating relaxation techniques and cognitive behavioral therapy (CBT), we managed to address the deep‐rooted anxiety and distress stemming from her situation. Maladaptive sexual beliefs and intrusive thoughts related to sexuality were reconstructed in a way that allowed the patient to regain a sense of control over her unwanted arousal, breaking the cycle of distress caused by distorted perception about sexual arousal and its implications. Hence, by seeing the effectiveness of the dual approach of pharmacotherapy and psychotherapy, we can conclude that a multidisciplinary treatment can substantially improve the outcomes for PGAD patients.

Additionally, other therapeutic protocols that are worth considering are pelvic floor physical therapy to address the possible pelvic muscular dysfunction that has been reported as a cause of PGAD [7], and sexual health education to develop a better mind–body connection. Consistent follow‐ups with both psychiatrists and gynecologists to make adjustments to the treatment plans based on patient's developing needs can potentially improve long‐term outcomes.

## 6 Conclusion

The case report concludes that PGAD is a multifactorial condition. While limited data exist on the correlation between its biopsychosocial etiologies, requiring further future research, our study found a blend of neurophysiological, psychological, and reproductive factors in play. This underscores the complexity of the etiology of the PGAD, hence demanding a comprehensive approach to its treatment. Despite limited success with previous interventions, our treatment plan of combining pharmacotherapy with Sodium Valproate and Risperidone alongside CBT proved to be effective in relieving the symptoms. Therefore, a multidisciplinary approach to target both physiological and psychological elements of the disease is recommended. Future research should be carried out to investigate the effectiveness of strategies involving physical therapy and mindfulness practice in enhancing the overall outcomes.

## Author Contributions

**Abdullah Hassan:** conceptualization, investigation, validation, writing – original draft. **Arooma sagheer:** validation, writing – original draft, writing – review and editing. **Muhammad Khubaib Ullah:** investigation, writing – original draft. **Habib Ullah:** conceptualization, methodology, writing – original draft. **Bilal Hassan:** validation, writing – original draft. **Sardar Noman Qayyum:** writing – original draft, writing – review and editing. **Rafiullah Hotak:** writing – original draft, writing – review and editing.

## Funding

The authors have nothing to report.

## Consent

Written informed consent was obtained from the patient for publication of this case report. A copy of the written consent is available for review by the Editor‐in‐Chief of this journal on request.

## Conflicts of Interest

The authors declare no conflicts of interest.

## Data Availability

The data used to support the findings of this study are included within the article.
